# Investigation of preoxygenation methods in cesarean surgeries with the oxygen reserve index

**DOI:** 10.15537/smj.2022.43.12.20220548

**Published:** 2022-12

**Authors:** Duygu Kocakulak, Gamze Küçükosman, Bengü Gülhan Köksal, Çağdaş Baytar, Rahşan D. Okyay, Keziban Bollucuoğlu, Tuğçe Öztürk, Özcan Pişkin, Hilal Ayoğlu

**Affiliations:** *From the Anesthesiology and Reanimation Department, Zonguldak Bülent Ecevit University, Faculty of Medicine, Zonguldak, Turkey.*

**Keywords:** Oxygen Reserve Index, preoxygenation, cesarean section

## Abstract

**Objectives:**

To investigate preoxygenation methods that were carried out for 3 minutes (min) at tidal volume and 30 seconds (s) with the 4 deep vital capacity technique using the Oxygen Reserve Index (ORI) among pregnant women.

**Methods:**

This prospective study was carried out between December 2020 and 2021. The patients were randomly divided into 2 groups with the provision of preoxygenation using 100% O_2_ at a rate of 10 L.min-1 for 3 min at normal tidal volume (Group 1) and 30 s with the 4 deep vital capacity technique (Group 2). For the pregnant women who underwent routine anesthesia induction, hemodynamic parameters before preoxygenation, as well as their fraction of inspired O_2_ (FiO_2_), fraction of expired O_2_ (FeO_2_), and ORI values were recorded after preoxygenation and 0, 3 and 7 minutes after intubation (T1, T2, T3, and T4).

**Results:**

The study was completed with 66 patients. FiO_2_ values were found to be low in T1 (*p*=0.012) in Group 1, and high in FeO_2_ values in T1 and T2 (*p*=0.025 and 0.009) in Group 2, while no significant differences were found at other times (*p*>0.05). Oxygen Reserve Index values did not show a significant difference in comparisons between groups, but ORI values of Group 1 after intubation were significantly lower than those measured after preoxygenation in in-group comparisons (*p*<0.001). According to the results of the correlation analyses between the mean ORI values and their mean FeO_2_ and FiO_2_ values, there were weak and positive statistically significant relationships at T3 and T4 (*p*<0.05).

**Conclusion:**

As we obtained greater FiO_2_ and FeO_2_ values in preoxygenation with the 30 s 4 deep vital capacity method, and because this method did not cause a significant decrease in the post-intubation ORI values, we believe that the usage of this method in cesarean section surgeries may be appropriate.

**T**he term preoxygenation refers to the procedure of the alveolar exchange of the mixture of air inside the functional residual capacity (FRC) that contains nitrogen and water vapor with 100% oxygen (O_2_).^1^ Performing preoxygenation with high-fraction O_2_ before anesthesia induction and endotracheal intubation (ETI), increases the O_2_ reserve in the lungs, and it is a method that has been used and accepted for years because it delays the time of apnea-related desaturation development.^2^ While preoxygenation can be performed using different methods, the most frequently utilized method is to inspire 100% O_2_ for 2-10 minutes (min).^3-5^ In addition to this, other recommended methods include the 4 deep breaths/30 seconds method that provides equivalent oxygenation to deep inspiration and deep expiration, providing the inflation of atelectatic areas in the basal lungs to increase FRC.^1^ The efficiency of preoxygenation is determined based on the time from the onset of apnea to a certain lower limit of peripheral oxygen saturation (SpO_2_).^6^ Although it has been reported that SpO_2_ levels should be >93% to obtain sufficient preoxygenation in emergencies, this practice might not show the adequacy of preoxygenation.^7,8^ The end-tidal oxygen level is used prevalently in anesthesia, and when it is ≥90%, it is usually accepted as an indicator of adequate preoxygenation.^6^ On the other hand, it has been reported that a value of >85% can also show adequate denitrogenation and preoxygenation.^9,10^ It is known that by a 40% increase in the tidal volume and a 20% reduction in FRC in pregnant women, denitrogenation occurs faster than in the case of non-pregnant women.^11^ Studies comparing different preoxygenation techniques in pregnant women have reported that fractions of expired O_2_ (FeO_2_), varying in the range of 79-90%, provide sufficient preoxygenation.^12-14^

Tissue oxygenation in anesthesia apAplications is monitored through SpO_2_ and the partial pressure of oxygen in arterial blood.^15^ The oxygen reserve index (ORI) is a measurement technique derived from hemoglobin oxygenation measurement sensors that shows the O_2_ reserves in arterial blood and can instantly assess tissue oxygenation. It has been reported that ORI, which aims to provide information on the patient’s status in the moderately hypoxic range (100mmHg <PaO_2_ ≤200 mmHg) and is an index that has a unitless scale in the range of 0-1, shows an early warning for an imminent hypoxic state and can also allow the prediction of unwanted hyperoxia cases.^16-19^

In our study, we aimed to investigate preoxygenation methods that were carried out for 3 min at tidal volume and 30 seconds with the 4 deep vital capacity technique using the ORI among pregnant women.

## Methods

**Figure 1:**
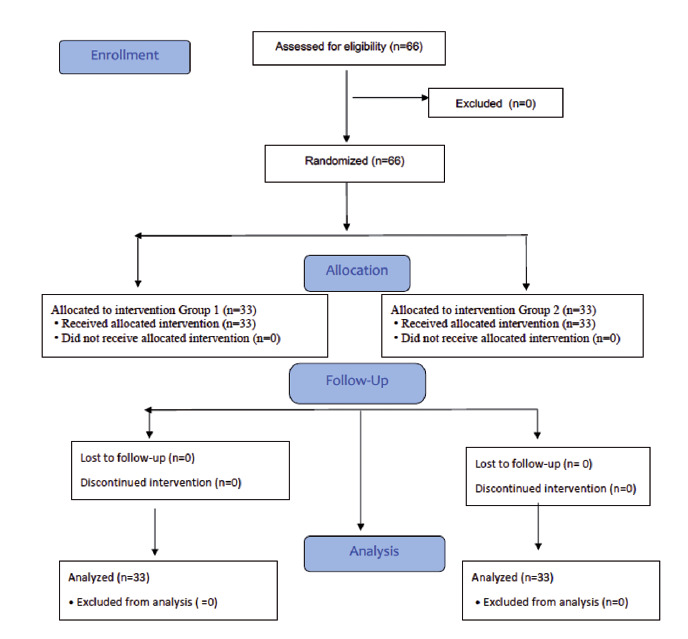
- Consolidated standards of reporting trials flow diagram of the study.

This study was carried between December 2020 and December 2021 after obtaining the approval of the ethics committee of the faculty (protocol number: 2020/22, ClinicalTrials.gov Identifier: NCT05395975) and the written consent of the patients. The Consolidated Standards of Reporting Trials (CONSORT) flow diagram was used for patient enrollment (Figure 1).^20^

Our prospectively planned study included 66 patients scheduled for cesarean surgery, whose operation time was approximately 30-90 min, who were in the American Society of Anesthesiologists (ASA) Status II risk class, aged 18-45 years, in their >36th gestational week, and did not agree to the use of regional anesthesia techniques. The sample excluded patients with preeclampsia, eclampsia, fetal distress, morbid obesity, a history of malignant hyperthermia, opioid sensitivity, alcohol or drug addiction, congestive heart failure, chronic obstructive pulmonary disease (COPD), coronary artery disease, significant anemia, liver and kidney diseases, hypovolemia, hypotension, sepsis, allergies to the drugs that were used in the study, or suspicion of a difficult airway.

The demographic characteristics, Mallampati scores, ASA risk scores, anesthesia times, and operative times of the patients were recorded. In addition to routine hemodynamic monitoring, the ORI sensor (ORi™, Masimo Corp., Irvine, CA, USA) was placed onto the fourth finger in the upper extremity, on which the blood pressure measurement cuff was not attached. The ORI sensor was wrapped to prevent exposure to light, and it was connected to the oximeter device (Root® platform Pulse CO-Oximetre, Masimo Corp., Irvine, CA, USA). The patients were given the left 15° lateral position to prevent aortocaval compression. In the patients who were not given premedication, vascular access was achieved with an 18–20 gauge peripheral venous catheter, and 0.9% saline infusion was administered at an infusion rate of 10 mL/kg/s. A 7-cm-high pillow was placed under the head of each patient.

The hemodynamic parameters of all patients before preoxygenation were recorded. The patients were informed on both preoxygenation methods, and they were told that a facemask would be placed tightly on their face, and that they would inhale 100% O_2_ from it. The patients who were randomized using the closed envelope method were divided into 2 groups for preoxygenation before anesthesia induction: Group 1 underwent preoxygenation at the normal tidal volume for 3 min with 100% O_2_ at a flow rate of 10 L.min-1; Group 2 underwent preoxygenation by being instructed to take a deep breath and exhale every 6-7 seconds (s) for 30 s (4 deep vital capacity [after maximum exhalation and maximum inhalation]) with 100% O_2_ at a flow rate of 10 L.min-1.

Apneic oxygenation was maintained for 60 s in patients who underwent rapid sequence induction following preoxygenation. All patients were intubated with 7–7.5 endotracheal tubes with introducers by anesthesia assistants who had at least 2 years of seniority and experience intubation with the McGrathTM MAC videolaryngoscope (Aircraft Medical Ltd., Edinburgh, UK) (at least 20 experiences). The accuracy of intubation was tested by monitoring the passage of the endotracheal tube from the vocal cords and capnography detection. The intubation time (time measured from the entry of the blade part of the laryngoscope into the mouth of the patient to the passage of the endotracheal tube between the vocal cords) was recorded. Following endotracheal intubation, all patients were ventilated at a tidal volume of 8 ml.kg-1 and a respiratory rate of 12 min-1. Anesthesia maintenance was achieved by providing a mixture of 4 L.min-1 50/50%: O_2_/air and 2% sevoflurane (Forane, Abbott Lab., England). After the delivery of the infant, an intravenous (IV) bolus of 1 mcg.kg-1 fentanyl (Fentanyl Citrate, Abbott Lab. North Chicago, USA) and 20 units of oxytocin (Synpitan Forte, Deva Holding A.Ş., Küçükçekmece/ Istanbul) infusion were administered.

The parameters in our study (mean arterial pressure [MAP], heart rate [HR], SpO_2_, inspired O_2_ fraction [FiO_2_], FeO_2_ and ORI) were recorded after preoxygenation and 0, 3 and 7 minutes after intubation (T1, T2, T3, and T4).

We planned to administer IV 5-10 mg ephedrine (Efedrin Hidroklorür Biosel 0.05g/1ml ampul, OSEL İlaç San. ve Tic. A.Ş Beykoz/ Istanbul) when the MAP value of each patient decreased by >20% compared to their control value, IV 0.5 mg atropine (Atropin Biosel 0.5 mg ampul, OSEL İlaç San. ve Tic. A.Ş Beykoz/ Istanbul) when their HR fell below 50 beats.min-1, and ventilate the patient with 100% O_2_ when their SpO_2_ value dropped under 93%.

All patients were administered 10 mg.kg-1 IV paracetamol (Parol 10 mg/ml, Atabay Kimya San. Ve Tic. A.Ş, Kadıköy/ İstanbul) infusion for postoperative pain and 10 mg IV metoclopramide (Vomepram 10mg/2ml, VEM İlaç San. ve Tic. Ltd. Şti., Çankaya/ Ankara) for nausea-vomiting. After the last skin suture, the anesthetic agents were stopped, and the patients were ventilated manually with 100% O_2_ until their spontaneous breathing returned. The routine reanimation protocol was applied to the patients, and following their extubation, they were taken to the recovery unit.

### Statistical analysis

The data were analyzed using the Statistical Package for the Social Sciences, version 23.0 (IBM SPSS Inc. Chicago, IL, USA) program. Conformity with normal distribution was analyzed using the Kolmogorov-Smirnov test. In the comparisons of 2 independent groups, independent-samples t-tests were used for the normally distributed variables, while the Mann-Whitney U test was used for the non-normally distributed variables. The Friedman test was used to compare the non-normally distributed variables against time for both groups, and multiple comparisons were made using the Dunn test. Spearman’s Rho correlation analysis was carried out to analyze the relationships between quantitative data. Pearson’s Chi-squared test and Yates correction were utilized to compare the categorical data based on the groups. The results of the analyses for the quantitative data are presented as mean ± standard deviation (x ± SD) and median (minimum-maximum). Considering the mean ORI values, in a 95% confidence interval (1- α), at a 95.2 % testing power (1- β), and an effect size of d=0.822, it was determined that the sample should include 66 cases in total, 33 in each group.^15^ A *p*-value of <0.05 was considered significant.

## Results

Our study was completed with 66 patients, and no patients were excluded from the study. There was no statistically significant difference between the 2 groups in terms of the demographic characteristics of the patients, their Mallampati scores, intubation times, operative times, or anesthesia times (*p*>0.05; Table 1).

**Table 1:** - Comparison of patients’ demographic data, Mallampati scores, intubation, surgery, and anesthesia times.

Characteristics	Group 1 n (33)		Group 2 n (33)		*P*-value
	Mean±SD	Median (min–max)	Mea±SD	Median (min–max)	
Age (year)	31.00±6.16	32 (19-41)	30.52±5.57	31 (19-40)	0.739**
Weight (kg)	80.42±16.98	81 (51-110)	79.67±12.92	78 (58-105)	0.839**
Length (m)	1.64±0.06	1.65 (1.51-1.75)	1.64±0.06	1.63 (1.52-1.75)	0.938*
BMI (kg/m^2^)	29.77±5.48	29.76 (21.39-38.06)	29.49±3.91	30 (21.26-35.86)	0.812**
Mallampati score (I/II/III)	9/16/8		13/12/8		0.306*
Intubation time (second)	13.21±4.47	12 (6-22)	13.79±5.69	12 (6-30)	0.974*
Operation time (minute)	49.58±9.63	50 (30-75)	52.48±9.42	50 (40-75)	0.250*
Anesthesia time (minute)	58.42±10.61	57 (35-90)	63.27±12.50	60 (45-90)	0.113*

*Mann-Whitney U test, **Independent-samples t-tests, SD: standard deviation, Min: minimum, Max: maximum, Group 1: 3 minutes at normal tidal volume, Group 2: 30 seconds with the 4 deep vital capacity technique, BMI: body mass index

The intergroup comparisons of the FiO_2_ values showed a significant difference between the groups only after preoxygenation (T1) (*p*=0.012) and no significant difference at other measurement times (*p*>0.05; Table 2). Both groups showed statistically significant intragroup differences in terms of their FiO_2_ values based on measurement times (*p*<0.001; Table 2). The FiO_2_ values of the patients in both groups started to decline after preoxygenation, and the lowest FiO_2_ values in both groups were measured at the 7th min after intubation.

**Table 2:** - Comparison of in-group and between-group fraction of expired oxygen values.

Time	Group 1 n (33)		Group 2 n (33)		*P*-value*
	Mean±SD	Median (min–max)	Mean±SD	Median (min–max)	
T1	91.97±3.5	92 (81–97)c	93.85±4.67	95 (82–99)b	0.012
T2	90.33±5.42	92 (74–97)c	90.24±5.41	91 (78–98)b	0.908
T3	58.94±5.52	60 (49–69)b	57.15±4.36	58 (49–64)a	0.081
T4	55.39±4.77	58 (46–60)a	55.18±4.18	57 (46–60)a	0.287
*P*-value^†^	<0.001		<0.001		

Group 1: 3 min at normal tidal volume, Group 2: 30 s with the 4 deep vital capacity technique. SD: standard deviation, Min: minimum, Max: maximum, T1: after preoxygenation, T2: immediately after intubation, T3: at the 3rd min after intubation, T4: at the 7th min after intubation, ^*^ *P*-value: Comparison between groups. ^†^ *P*-value: Compared within the group, a-c: There is no difference between times with the same letter in a group.

The intergroup comparisons of the FeO_2_ values showed significant differences between the groups only at T1 and T2 (*p*=0.025 and 0.009) and no significant difference at other measurement times (*p*>0.05; Table 3). Both groups showed statistically significant intragroup differences in terms of their FeO_2_ values based on measurement times (*p*<0.001; Table 3). As in the FiO_2_ values, the FeO_2_ values of the patients in both groups started to decline after preoxygenation, and the FeO_2_ values were the same in both groups at T4 (Table 3).

**Table 3:** - Comparison of fraction of expired oxygen values within and between groups.

Time	Group 1 n (33)		Group 2 n (33)		*P*-value*
	Mean±SD	Median (min.–max.)	Mean±SD	Median (min.–max.)	
T1	81.64 ± 7.07	83 (62–91)c	84.27 ± 9.89	88 (56–95)b	0.025
T2	76.27 ± 6.54	77 (66–89)c	80.85 ± 8.34	82 (62–98)b	0.009
T3	53.70 ± 6.07	54 (42–65)b	51.58 ± 3.86	51 (45–60)a	0.086
T4	49.21 ± 4.24	50 (41–54)a	49.30 ± 3.95	50 (41–54)a	0.732
*P*-value^†^	<0.001		<0.001		

* Mann-Whitney U test; ^†^Friedman test, SD: standard deviation; Min: minimum; Max: maximum, Ggoup 1: 3 min at normal tidal volume, Group 2: 30 s with the 4 deep vital capacity technique, T1: after preoxygenation, T2: immediately after intubation, T3: at the 3rd min after intubation, T4: at the 7th min after intubation, ^*^ *P*-value: Comparison between groups. ^†^ *P*-value: Compared within the group, a-c. There is no difference between times with the same letter in a group.

There was no significant difference between the ORI values of the 2 groups at any measurement time (*p*>0.05; Table 4). In the intragroup comparisons, both groups showed statistically significant differences in terms of their ORI values based on time (*p*<0.001; Table 4). The highest ORI values in both groups were measured after preoxygenation, while these values showed a decreasing trend in later measurements (Table 4).

**Table 4:** - Comparison of in-group and between-group oxygen reserve index values.

Time	Group 1 (n: 33)		Group 2 (n: 33)		*P*-value*
	Mean±SD	Median (min.–max.)	Mean±SD	Median (min.–max.)	
T1	0.52±0.23	0.58 (0.09–1)b	0.47±0.33	0.42 (0–1)b	0.342
T2	0.27±0.20	0.29 (0–0.71)a	0.39±0.31	0.33 (0–1)ab	0.173
T3	0.20±0.22	0.12 (0–0.62)a	0.24±0.22	0.25 (0–0.67)a	0.427
T4	0.15±0.17	0.17 (0–0.48)a	0.23±0.24	0.20 (0–0.9)a	0.226
*P*-value^†^	<0.001		<0.001		

* Mann-Whitney U test, ^†^Friedman test, SD: standard deviation, Min: minimum, Max: maximum, Group 1: 3 min at normal tidal volume, Group 2: 30 s with the 4 deep vital capacity technique, T1: after preoxygenation, T2: immediately after intubation, T3: at the 3rd min after intubation, T4: at the 7th min after intubation, ^*^ *P*-value:comparison between groups. ^†^ *P*-value: Compared within the group, a-b: There is no difference between times with the same letter in a group.

According to the results of the correlation analyses between the ORI values of all patients and their FeO_2_ and FiO_2_ values, there were weak and positive statistically significant relationships for the values obtained at the 3rd and 7th min after intubation (Table 5).

**Table 5:** - Correlation analysis between ORI and FeO_2_ and FiO_2_.

Time	ORİ	Coefficient	FeO_2_	FiO_2_
T1	ORİ	r	0.031	0.113
		p	0.805	0.366
T2	ORİ	r	0.189	0.155
		p	0.129	0.214
T3	ORİ	r	0.246	0.270
		p	0.047	0.028
T4	ORİ	r	0.390	0.372
		p	0.001	0.002

r: Spearman’s Rho correlation coefficient, T1: after preoxygenation, T2: immediately after intubation, T3: at the 3rd min after intubation, T4: at the 7th min after intubation, ORI: oxygen reserve index, FeO_2_: fraction of exhaled O_2_, FiO_2_: Fraction of inspired O_2_

## Discussion

In our study, we investigated different preoxygenation techniques (3 min of tidal volume and 30 s 4 deep breaths with 100% O_2_) in pregnant women using ORI, and we obtained similar ORI values with both methods, while greater FiO_2_ and FeO_2_ values were observed after preoxygenation with the 30 s 4 deep vital capacity method. We believe that the use of this method in cesarean section surgeries may be more appropriate.

Chiron et al^12^ compared 3 different preoxygenation techniques (30 s 4 deep vital capacity at a flow rate of 9 L.min-1 with O_2_, 1 min 8 deep vital capacity at 15 L.min-1 with O_2_, and 3 min tidal volume at 9 L.min-1 with O_2_) in pregnant women, and reported higher rates of FeO_2_ >90% in the 1 min 8 vital capacity and 3 min tidal volume respiration methods, arguing that the 1 min 8 deep vital capacity method to be more appropriate for patients undergoing rapid sequence induction at emergency obstetrics services. Norris et al.^21^ reported that in patients scheduled for cesarean surgery, performing preoxygenation with the 30 s 4 vital capacity method can raise PaO_2_ levels to a degree equivalent to that of applying 100% O_2_ for 3–5 min at a normal tidal volume. Considering the relationship between oxygen reserve index and PaO_2_; The similarity of ORI values in both preoxygenation methods in our study suggests that PaO_2_ values may also be similar. In this study, we determined that the patients who underwent preoxygenation with 30 s of 4 deep breaths had higher FiO_2_ and FeO_2_ values following preoxygenation. We considered that short preoxygenation caused a more effective respiratory pattern, whereas long preoxygenation could have shown negative effects on respiratory capacity by causing anxiety and fatigue. We believe that further studies are needed on this subject.

It has been reported that the time required for the lungs to denitrogenize during preoxygenation is shorter due to decreased FRC in pregnant women and they desaturate more rapidly during apnea.^22,23^ The method of applying 100% O_2_ with a tidal volume of 3-5 min, which is commonly used for preoxygenation, may not be practical in some obstetric emergencies due to time constraints.^21,24^ Bernard et al^24^ compared the practice of the 30 s 4 deep breaths method and the 4 min tidal volume method with 100% O_2_ at a flow rate of 10 L.min-1 in 27 patients undergoing general anesthesia (GA) in cesarean surgery and found no significant difference between the desaturation times (until SpO_2_ declined to 93%) of the groups. Moreover, in their study that included 1,050 patients, Baillard et al^25^ determined FeO_2_ values of <90% in 56% of the patients who underwent preoxygenation with 100% O_2_ at for 3 min at tidal volume, and they reported that this result could be associated with difficult mask ventilation, and this method resulted in insufficient preoxygenation. In our study, where 77.3% of the patients showed FeO_2_ values of <90% at the end of preoxygenation, this rate was 91% in Group 1 and 64% in Group 2. Therefore, compared to the literature, it may be stated that both preoxygenation methods were insufficient in the pregnant women who participated in our study. We believe that 30 s 4 deep vital capacity technique with preoxygenation method may be more appropriate in ASA II cases who are scheduled for emergency cesarean section. Additionally, we believe that there is a need for further studies on the adequacy of 90% FeO_2_ in preoxygenation for pregnant women with comorbidities.

In alveolar preoxygenation, the aim is to achieve FeO_2_ ≥90%.^26^ In a multicenter study (n=2,398), Baillard et al.^27^ reported that factors such as hypertension, COPD, expected difficult mask ventilation/intubation, and emergency surgery were risk factors associated with hypoxemia (n=158) after preoxygenation. In the same study, the FeO_2_ values after preoxygenation were determined as <90% in 713 patients, and by defining this as difficult preoxygenation, the authors reported that this was associated with risk factors for hypoxia. In our study, although we determined FeO_2_ <90% after preoxygenation in 77.3% (n=51) of our patients, we observed that their SpO_2_ values did not drop below 93%. We believe that our patients did not develop hypoxia because they did not have the risk factors associated with hypoxia.

Tsymbal et al^15^ in their studies in which they performed induction/ETE after the ORI reached the plateau level in preoxygenation in obese (30<BMI<40) and patients with normal (19<BMI<25) BMI; They applied apneic oxygenation until SpO_2_ reached 94% and recorded both pulse oximetry and ORI alarm times until SpO_2_ decreased from 97% to 94% after intubation. They showed that ORI provided clinically significant additional warning times for both obese patients and patients at normal weights. In their study that aimed to evaluate preoxygenation with ORI in daily anesthesia monitoring, Cheng et al^28^ compared ORI, SpO_2_, and PaO_2_ decrease trends in 25 patients who underwent GA until their SpO_2_ values declined to 90%, and reported that ORI provided a warning 145 s earlier than SpO_2_ did. In our study, where we continued apneic oxygenation until intubation following anesthesia induction, we determined a significant decrease in the ORI values of Group 1 from the measurement made after preoxygenation to that made at the end of intubation. On the other hand, in Group 2, there was no significant difference between the ORI values measured after preoxygenation and those measured at the end of intubation. This suggests that preoxygenation with the 30 s 4 deep breaths method can be safer in pregnant women.

### Study limitations

The limitations of our study included the fact that we excluded ASA ≥ III patients, expected difficult airway cases, and patients with problems in their pulmonary mechanics. In addition, we did not study PaO_2_, did not obtain data about smoking status, and did not investigate anxiety levels.

In conclusion, as we obtained greater FiO_2_ and FeO_2_ values in the preoxygenation of the pregnant women who participated in our study with the 30 s 4 deep vital capacity method using 100% O_2_, and because this method did not cause a significant decrease in the post-intubation ORI values, we believe that the use of this method in cesarean section surgeries may be appropriate. In our opinion, preoxygenation procedures performed under the guidance of the ORI are highly important for patient safety, and there is an urgent need for more studies on this topic.
